# A mini review on advances in diagnostic techniques for *Schistosoma japonicum* detection and its epidemiological features among humans and wild rodents in China

**DOI:** 10.3389/fvets.2026.1857648

**Published:** 2026-05-28

**Authors:** Fen Chen, Xi Hu, Qi Xiao, Lei Tan, Wei Liu

**Affiliations:** 1 Xiangnan University, Chenzhou, China; 2 College of Animal Science and Technology, Yangtze University, Jingzhou, China; 3 Yueyang Animal Disease Prevention and Control Centre, Yueyang, China; 4 Research Center for Parasites and Vectors, College of Veterinary Medicine, Hunan Agricultural University, Changsha, China

**Keywords:** China, detection method, epidemiological characteristics, *Schistosoma japonicum*, various host species

## Abstract

Schistosomiasis is a zoonotic parasitic disease caused by *Schistosoma japonicum* infection, representing a significant public health concern for both animals and humans in China. A thorough understanding of the epidemiological features and diagnostic techniques associated with schistosomiasis is crucial for conducting prevention, control, and eradication strategies. This review provides a comprehensive update on the current diagnostic technologies and the prevalence of *S. japonicum* in human and wild rodent populations in China, focusing on literature published from 2015 to the present. In general, diagnostic methods include morphological identification, serological assays, and molecular techniques. While traditional methods like Kato-Katz remain widely used, emerging tools such as PCR-based assays, isothermal amplification (LAMP, RPA, RAA), and CRISPR/Cas systems offer enhanced sensitivity and suitability of field deployment. Serological tests (IHA, ELISA) are valuable for large-scale screening but face specificity challenges. A descriptive aggregation of 37 studies involving 46,910,186 human serum samples revealed an overall seroprevalence rate of 1.54% (95% CI: 1.53–1.54), with significant variation across 10 provinces (0.08% in Fujian to 4.95% in Yunnan). Higher seroprevalence was observed in males, local residents, and individuals engaged in farming or fishing. Concurrently, a narrative synthesis of 24 studies across seven provinces showed a substantially higher prevalence of 8.97% (95% CI: 8.50–9.44) in 14,381 wild rodents, with *Rattus norvegicus* showing the highest infection rate (37.44%). In conclusion, despite significant control progress, *S. japonicum* remains endemic in specific regions, with wild rodents serving as critical reservoir hosts. Integrating sensitive molecular diagnostics into surveillance programs and targeting rodent reservoirs are essential for achieving the national goal of schistosomiasis elimination by 2030.

## 1 Introduction

Schistosomiasis is a significant zoonotic parasitic disease caused by infection with *Schistosoma japonicum* (*S. japonicum*), which is widely distributed across numerous countries, predominantly in tropical and subtropical regions (1). Based on global burden assessment of schistosomiasis, transmission of the disease has been reported in 79 countries, it is estimated that more than 253.7 million individuals require targeted preventive treatment in 2024 (2). In China, schistosomiasis is classified as a legally recognized Class B infectious disease and remains primarily endemic in several provinces and autonomous regions along the middle and lower reaches of the Yangtze River, which presents a significant threat to public health and impedes social and economic development (3). At present, schistosomiasis remains a primary focus of parasitic disease prevention and control in China (4).

**Figure 1:**
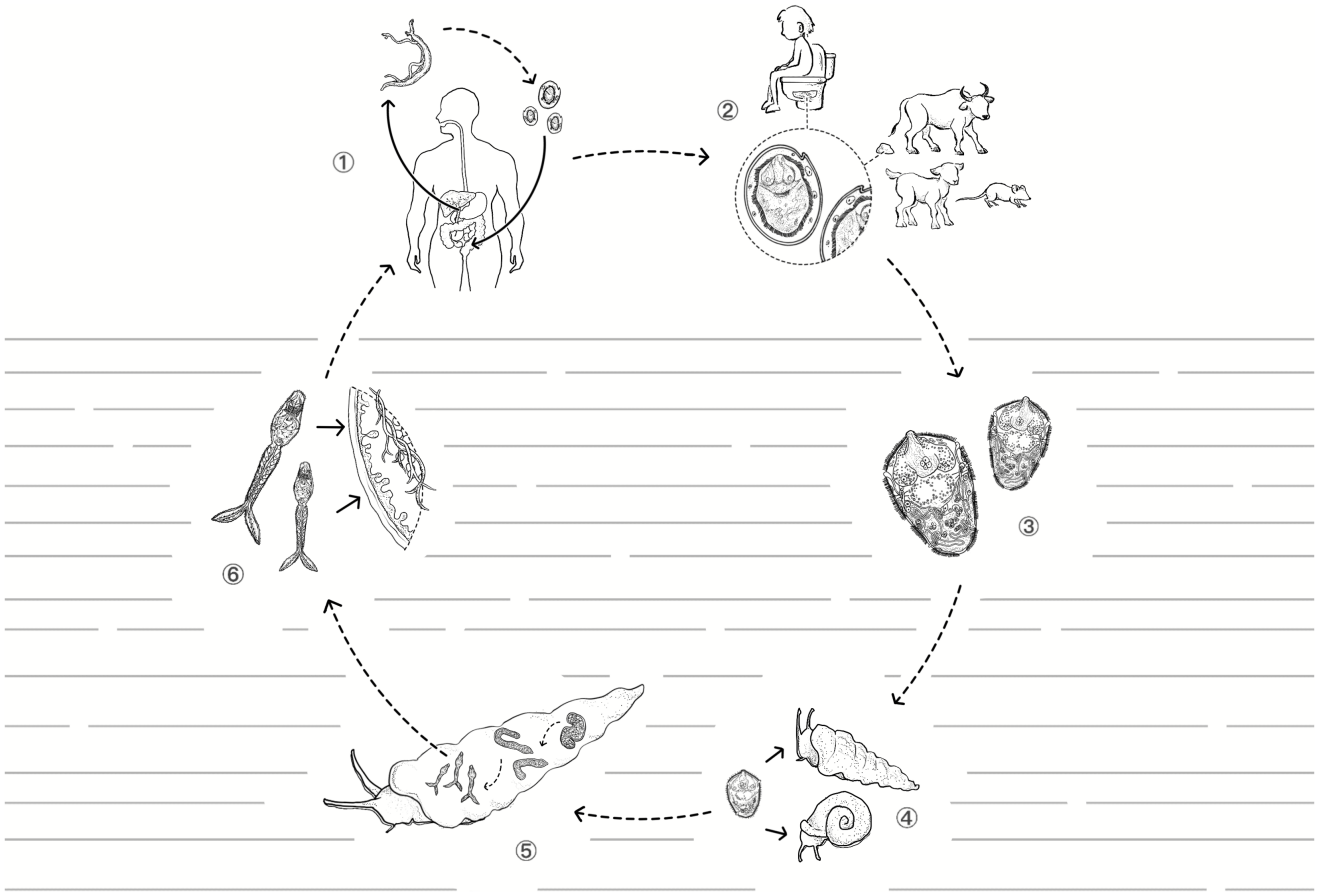
The life cycle of the adult *S. japonicum* encompasses multiple distinct stages, among which, the definitive host for this parasite included humans, cows, goats, and wild rodents.

*S. japonicum* is capable of infecting a wide range of mammalian hosts, and exhibits a complex life cycle (5). As shown in Figure 1, the life cycle of *S. japonicum involves* egg excreted in feces, hatching into miracidia that infect snails, where they develop into cercariae. Cercariae are released into water and actively penetrate the skin of definitive hosts, then migrate via the bloodstream to the mesenteric veins and liver, maturing into egg-producing adult worms (6). Prior research has established a strong association between schistosomiasis and a range of pathological conditions, including hepatosplenic disease (7), urinary tract inflammation, urticaria, chronic pulmonary disorders, and pulmonary arterial hypertension (7–9). Additionally, schistosomiasis has been shown to impair the efficacy of certain disease vaccines (10). Early diagnosis is essential for the prevention and control of schistosomiasis, its complex biological features directly underlie the major diagnostic challenges in chronic *S. japonicum* infection, including low fecal sensitivity and reliance on serology or molecular methods.

Schistosomiasis has persisted in China for over two millennia (11). During the 1950s, the disease was extensively prevalent throughout the Yangtze River Basin and was once regarded as a significant public health threat. To effectively prevent and control this disease, China has recently implemented a series of comprehensive interventions. These measures include the chemical and physical eradication of snail vectors, pharmacological treatment of infected individuals, modification of lifestyle in endemic regions, and the promotion of public awareness and education. Although the epidemic has been largely controlled in most areas, *S. japonicum* continues to pose a risk to public health in certain localities. Therefore, timely and accurate diagnostics enable early treatment, reduce transmission, and support targeted mass drug administration. Additionally, understanding the prevalence of schistosomiasis across various host species is essential for effective disease management and environmental remediation efforts.

Considering the aforementioned considerations, this review provides an updated synthesis of current knowledge regarding *S. japonicum* in China. Unlike previous meta-analyses that systematically quantified the epidemiological characteristics of *S. japonicum* in human populations (1982–2020) (1) and in wild rodent populations (1982–2020) (12). This review offers a qualitative, interpretive synthesis of studies published from January 1, 2015 to April 1, 2026, with a particular focus on comparing epidemiological patterns before and after the COVID-19 pandemic. This temporal focus and narrative approach allow for the integration of contextual factors, such as pandemic-related disruptions to healthcare systems and human-environment interaction (13, 14) as well as the potential impact of vaccine-induced immune modulation on the prevalence of *S. japonicum* infection in human populations (15, 16). Moreover, Chen et al. (17) previously reviewed diagnostic techniques for detecting *S. japonicum* infection. The present review highlights recent methodological advancement developed since 2015, including novel detection tools that have emerged in the post-pandemic period. By providing a comprehensive overview, qualitative overview with an emphasis on recent temporal dynamics and diagnostic innovations, this review complements and extends existing quantitative meta-analyses, and serves as a valuable resource to support future initiatives targeting the prevention, control, and potential eradication of this parasitic disease.

## 2 The history of *S. japonicum* in China

*S. japonicum* has been endemic in China for over two millennia, as demonstrated by the discovery of its eggs in two ancient human remains dating back approximately 2100 years (18). The initial documented case of *S. japonicum* infection in humans, along with the initial characterization of its eggs, was reported in China in 1905 (19). Shortly after, cases were documented in other provinces within a relatively short period. Notably, Mao conducted a comprehensive review of *S. japonicum* in China prior to 1948, revealing that this parasite was mainly endemic in areas surrounding Dongting Lake, Poyang Lake, and Tai Lake. In these areas, the average infection rate exceeded 21.00%, with over 5 million individuals affected (20). Given the severe health risks to individuals and the considerable economic burden on families, the disease caused by *S. japonicum* was historically regarded as a devastating affliction in China (21).

Following the establishment of the People’s Republic of China, the central government and local authorities have prioritized the prevention and control of schistosomiasis. Strong governmental leadership, an effective operational framework, extensive public participation, sustained implementation of control measures, and continuous advancement in strategic approaches and scientific technologies have collectively facilitated significant advancements in the prevention and control of schistosomiasis within China (22). For instance, the seroprevalence of *S. japonicum* among human populations in China has decreased significantly, from 38.4% in 1984 to 2.4% in 2020 (1). In order to promote the development of a healthy China and protect public health, the State Council promulgated the “Health China 2030” Planning Outline in 2016, which established the objective of eradicating schistosomiasis in all endemic counties nationwide by the year 2030 (23).

## 3 Current diagnostic technologies for detecting *S. japonicum*

At present, *S. japonicum* continues to be endemic in certain provinces and regions of China, posing a significant public health challenge (1). In light of this, early diagnosis is critical in alleviating the adverse effects of this parasitic infection and minimizing the treatment expenses incurred by affected individuals. In addition to traditional consultation, researchers have developed a range of detection methods to monitor the prevalence of *S. japonicum* across diverse host species. These diagnostic techniques can be broadly divided into three groups according to the detection targets and signal sources: morphological identification involving detecting different developmental stages of *S. japonicum* (24–26); molecular techniques that probe specific DNA sequences of the parasite (27–50); and serological methods which are designed to detect antibodies specific to *S. japonicum* (51–60). In this section, we offer an overview of the main characteristics of these diagnostic methods, which are also briefly summarized in Table 1.

**Table 1:** The main characteristics of various diagnostic approaches for detecting *S. japonicum*.

Methods	Targets (reference)	Operation period	Detection limit	Costs	Main characteristics
Kato-Katz	Eggs (24–26)	< 1 h	low	*	Kato-Katz technique and miracidium hatching test have been widely employed for the detection of *S. japonicum* infection across diverse clinical and field settings in China, which exhibit 100% specificity, while their sensitivities are relatively low.
Miracidium hatching test	Miracidium (24–26)	2 ~ 6 h	Higher than Kato-Katz method	***	
PCR	SjR2 gene (27)	1 ~ 3 h	0.5 eggs/g of stool	**	PCR method exhibited greater sensitivity than the conventional Kato-Katz and miracidium hatching test methods; however, its implementation requires specialized equipment and trained personnel.
Nested PCR	SjR2 gene (28)	> 3 h	One egg	***	Nested PCR demonstrated superior sensitivity compared with conventional PCR; however, it entails greater procedural complexity and higher associated costs.
Real-time PCR	SjTR1 gene (29)	1 ~ 2 h	200 ag of genomic DNA	**	Real-time PCR exhibited greater sensitivity than conventional morphological identification and PCR methods, which require less time but more expensive equipment.
Multiplex real-time PCR	- (31)	1 ~ 2 h	2.55 copies/μL of plasmid	***	Which has the capability to concurrently detect the presence of *S. japonicum* as well as other pathogens.
DdPCR	SjR2 and nad1 genes (32)	4 ~ 5 h	One egg; 0.05 fg of genomic DNA	****	DdPCR methods can accurately quantify the copy number of *S. japonicum* in different clinical samples.
	SjG28 gene (33)	4 ~ 5 h	38.94 ~ 194.74 copies/μL		
LAMP	SjR2 gene (35)	< 1 h	0.08 fg of genomic DNA	****	These method demonstrated higher sensitivity than conventional PCR, without requiring sophisticated equipment or complex procedures. However, their relatively high false-positive rate and high cost may limit broader application.
RAA	SjG28 gene (38)	37 °C for 30 min	20 copies/μL	****	
RAA combined with a nucleic acid dipstick test	SjG28 gene (39)	39°C for 15 min	10 copies/μL of plasmid and 1 pg. of genomic DNA	*****	
RPA	Sj28S gene (40)	39°C for 20 min	100 fg of genomic DNA and 100 copies/μL of plasmid	****	
RPA combined with LFD method	SjCHGCS19 gene (41)	39°C for 20 min	10^−6^ ng of genomic DNA	*****	
RPA integrated with a real-time DNA detection system	SjR2 gene (42)	39°C for 20 min	0.9 fg of target DNA	*****	The detection results of this novel assay showed good concordance with the Kato-Katz method.
RPA combined with CRISPR/Cas12a system	SjCHGCS20 gene (44)	40 °C for 30 min	10^−5^ ng of genomic DNA	*****	The detection outcomes can be monitored through blue light excitation, allowing for observation with the unaided eye.
LAMP combined with CRISPR/Cas12a system	Sj28S gene (45)	65 °C for 1 h	10 pg./μL for the genomic DNA and 1 copy/μL for the standard plasmid	*****	This approach demonstrated a reduction in false positive rates and an enhancement in sensitivity relative to the LAMP method.
RPA combined with CRISPR/Cas13a technique	Sjcox1 gene (46)	37 °C for 1 h	0.2 pg./μL for the genomic DNA	*****	This novel detection system provided a highly sensitive test that could be applied across diverse diagnostic settings
NGS	- (50)	Several days	High sensitivity	******	This approach can be utilized for the identification of unknown pathogens; however, it is associated with significantly greater costs and longer processing times compared to the aforementioned methods.
IHAT	- (52)	1.5 ~ 2.0 h	Relatively higher than Kato-Katz method	**	IHAT demonstrated higher sensitivity relative to the Kato-Katz and hatching tests, its specificity was comparatively lower.
Indirect ELISA	Cathepsin B antibody (57)	1.5 ~ 3.0 h	Higher than IHAT technique	***	These methods specifically detected anti-*S. japonicum* antibodies and showed greater sensitivity than IHAT. Notably, these methods have been frequently used for investigating the seroprevalence of this pathogen in human populations.
Double antibody sandwich ELISA	Sj29 antibody (58)	3.0 ~ 4.0 h		***	
Fluorescence immunochromatographic assay strip	Soluble egg antigen antibody (59)	10 ~ 30 min	1:10000 serum dilution of positive serum sample	***	These method exhibited a high concordance with ELISA and Kato-Katz techniques, which was suitable for on-site testing.
Gold immunochromatographic assay strip	Saposin antibody (60, 61)	10 ~ 30 min	1: 20480 serum dilution of positive serum sample	***	

The symbols “*,” “**,” “***,” “****,” “*****,” and “******” represent the relative expenses of the respective detection techniques, with costs increasing from low to high.

### 3.1 Morphological identification methods

Morphological identification techniques are regarded as the definitive standard for detecting various developmental stages of *S. japonicum* in tissue, water, and fecal specimens. Several diagnostic methods exist for the morphological identification of *S. japonicum* in fecal and water samples, including the direct smear method, the miracidium hatching test, and the Kato-Katz technique for egg detection, as well as dynamic automated identification systems for the detection of miracidia. Among these, the miracidium hatching test and Kato-Katz method have been widely employed for pathogen detection in clinical fecal samples, these techniques exhibit 100% specificity, while their sensitivities are relatively low (24). As for the morphological identification techniques employed for this parasite in definitive hosts, predominantly wild rodents, several methods are utilized. In addition to the Kato-Katz and miracidium hatching test methods, microscopy examination of mouse liver tissues, mouse mesenteric tissues, and mouse liver tissue homogenates is also conducted. Among these diagnostic approaches, microscopy analysis of mouse liver tissues and liver tissue homogenates demonstrated significantly higher detection rates compared to the other methods (25). For example, Xu et al. (25) conducted a comparative analysis of the efficacy of various etiological methods for detecting *S. japonicum* infection in wild mice. The detection rates of *S. japonicum* infection utilizing microscopy of mouse liver tissues, microscopy of liver tissue homogenates, the Kato-Katz technique, and the miracidia hatching test were 16.79% (286/1417), 11.04% (188/1515), 2.70% (46/1657), and 2.70% (46/1657), respectively.

### 3.2 Molecular detection methods

Although morphological identification techniques have been extensively utilized for the detection of *S. japonicum* (26). In recent years, various molecular detection techniques have been created for the early diagnosis of *S. japonicum* infection in clinical specimens. These approaches could be categorized into three main groups: ① conventional polymerase chain reaction (PCR) (27) and its advanced variants, including nested PCR (28), real-time PCR (29, 30), multiplex real-time PCR (31), and droplet digital PCR (ddPCR) (32, 33); ② isothermal amplification techniques, such as loop-mediated isothermal amplification (LAMP) (34–37), recombinase-aided isothermal amplification (RAA) assays (38, 39), and recombinase polymerase amplification (RPA) (40–42); and ③ CRISPR/Cas-based detection systems, which encompass CRISPR/Cas12a combined with LAMP or RPA method (44, 45), as well as CRISPR/Cas13a integrated with RPA techniques (46).

#### 3.2.1 Conventional PCR techniques

Of note, PCR techniques have been employed for both the early detection and genetic variation analysis of *S. japonicum* (25). However, nested PCR and real-time PCR methods exhibit greater sensitivity compared to conventional PCR approaches, rendering them more appropriate for the early diagnosis of this parasite in clinical specimens (28–30). For instance, Halili et al. (29) established a real-time PCR diagnostic assay targeting the *Schistosoma japonicum* Tandem Repeat 1 (jTR1) gene of *S. japonicum*, and demonstrated a detection limit of 200 ag of *S. japonicum* genomic DNA and one egg per gram of human stool. This sensitivity was comparable to that of the nested PCR method and markedly superior to previously reported PCR techniques (27). Moreover, the advancement of multiplex real-time PCR has enabled the simultaneous detection of *S. japonicum* and other pathogens, significantly reducing diagnostic costs (31). Morphological similarities and comparable movement patterns have been observed between *S. japonicum* and *Orientobilharzia*, and these two parasites also co-occur in certain geographic areas (31). Tang developed a multiplex real-time PCR method capable of simultaneously detecting both species, achieving a detection limit of 2.55 copies/μL for *S. japonicum* (31).

#### 3.2.2 Isothermal amplification techniques

##### 3.2.2.1 Lamp

In addition to these traditional PCR methodologies, the characteristics of LAMP, RPA, and RAA techniques-namely, their comparable sensitivity to real-time PCR and the absence of a need for complex laboratory infrastructure-render them particularly well-suited for on-site field detection in low endemicity areas (34). For example, Xu et al. (35) established a LAMP method for the detection of *S. japonicum*, achieving a detection limit of 0.08 fg/μL, which represented a sensitivity enhancement of 10000-fold compared to the conventional PCR method. Notably, these novel detection methods operated under isothermal conditions, thereby facilitating their application in field-based diagnosis settings (36, 37).

##### 3.2.2.2 RAA

Zhao et al. (38) developed a RAA assay targeting the jG28 gene of *S. japonicum*, achieving a detection limit of 20 copies/μL for the standard plasmid and 0.5 ng for parasitic DNA. Notably, this novel detection protocol can be performed at 37 °C for 30 min, while the detection of the final results requires the use of a gel imaging system (38). To address this limitation, Ye et al. (39) established a RAA assay integrated with a nucleic acid dipstick test for the detection of *S. japonicum*. In brief, following RAA amplification, the products were added into a nucleic acid dipstick test to generate visual readouts, demonstrating detection limits of 1.0 pg./μL for genomic DNA and 10 copies/μL for the standard plasmid (39).

##### 3.2.2.3 RPA

Beyond LAMP and RAA, research efforts have also focused on RPA technologies to develop innovative detection methodologies (40–42). For example, Wang et al. (40) established a sensitive, specific, straightforward and rapid method for the detection of *S. japonicum* nucleic acid using the RPA technique. This assay operates at 39 °C for 20 min, and exhibits a detection limit of 100 copies/μL for plasmid DNA or 100 fg of genomic DNA without observed cross-reactivity (40). To establish a single-step detection approach, Deng et al. (41) combined RPA with lateral flow dipstick method to create a rapid visual method for specifically targeting *S. japonicum*. Notably, this method achieved a detection limit of 10^−6^ ng/μL of genomic DNA; however, it demonstrated cross-reactivity with *Schistosoma mansoni* (*S. mansoni*) and *Schistosoma haematobium (S. haematobium)* (41). This cross-reactivity represents a significant limitation, particularly given the frequent co-endemicity of *S. mansoni* and *S. haematobium,* and *S. japonicum* in certain geographic regions. Therefore, further efforts should prioritize the enhancement of primer specificity to effectively mitigate cross-reactivity.

In addition, Xing et al. (42) developed a RPA assay integrated with a real-time DNA detection system for detecting *S. japonicum*’s genomic DNA in fecal samples. Specifically, this novel assay achieved sensitivity comparable to that of conventional real-time PCR, with a detection limit of 0.9 fg of target DNA per reaction (42). Moreover, when evaluated against the Kato-Katz method as the reference standard, the RPA assay demonstrated 100% sensitivity and 96.4% specificity, substantially higher than those of both IHA and ELISA (42).

#### 3.2.3 CRISPR/Cas based detection systems

In comparison to conventional molecular methods, CRISPR/Cas-based detection techniques presents several distinct advantages (47). These include single-nucleotide specificity, isothermal signal amplification without thermocycling, results in < 1 h, easy reprogramming for new pathogens, and compatibility with lateral flow readouts, making it a superior point-of-care diagnostic platform (47). In recent years, detection systems based on CRISPR/Cas technology have emerged as innovative platforms for pathogen diagnosis (48), including the identification of *S. japonicum*. Xu et al. (44) developed a rapid visualization method by integrating RPA with the CRISPR/Cas12a system for the detection of *S. japonicum*. Specifically, the SjCHGCS20 gene sequences were amplified by RPA, the resulting amplification products were visually detected using the CRISPR/Cas12a assay. This method can be operated at 40 °C for 30 min, and achieves a detection limit of 10^−5^ ng/μL of genomic DNA, exhibiting no cross-reactivity with other trematode species (44). Using a comparable approach, Lin and his colleagues applied LAMP and CRISPR/Cas12a technologies to target the Sj28S gene sequence of *S. japonicum* (45). Importantly, the sensitivity of this innovative detection method reached a detection limit of 10 pg./μL for the genomic DNA and 1.0 copy/μL for the standard plasmid (45).

In addition to the CRISPR/Cas12a system, MacGregor and his team colleagues developed a novel diagnostic platform for the detection of *S. japonicum* and *S. mansoni* by integrating RPA with the CRISPR/Cas13a technique (46). Notably, the amplified products can be detected through either fluorescent or colorimetric readouts, thereby enabling versatile application across diverse diagnostic settings (46). Furthermore, this innovative approach exhibited greater sensitivity than the conventional real-time PCR assay, achieving concordance rates of 93–100% with the real-time method across all tested samples (46).

#### 3.2.4 Metagenomic next-generation sequencing (mNGS)

Metagenomic next-generation sequencing (mNGS) is an advanced high-throughput detection technique that has been extensively used for the identification of novel pathogens, pathogen genome sequencing, and other applications (48, 49). Liu et al. (50) used the mNGS technology to successfully detect *S. japonicum*-specific circulating DNAs (cDNAs) in the serum samples of experimentally infected rabbits. Furthermore, a LAMP-based CRISPR-Cas12a technology targeting these newly identified cDNAs was subsequently developed to facilitate the early diagnosis of schistosomiasis in both human and murine models (50). Wu et al. (51) employed mNGS technology to identify potential pathogens in formalin-fixed paraffin-embedded tissue samples obtained from four suspected human cases of *S. japonicum* infection in Yunnan Province. Their findings confirmed the presence of *S. japonicum* in these regions, which is traditionally considered non-endemic for this parasite.

### 3.3 Serological detection method

In addition to the aforementioned diagnostic techniques, a range of serological methods have been developed to monitor the seroprevalence of *S. japonicum* using serum samples. In the absence of an available vaccine, these approaches serve as valuable tools for distinguishing infected individuals from healthy subjects. Accordingly, numerous serological assays have been established to detect specific antibodies against *S. japonicum*, thereby facilitating the initial screening of positive cases.

#### 3.3.1 Indirect hemagglutination test (IHAT)

The indirect hemagglutination test (IHAT) for detecting *S. japonicum* is a serological method that offers practical value for community screening and epidemiological studies, though it cannot reliably differentiate active from past infection. When serum samples containing antibodies specific to *S. japonicum* are introduced, these antigen-coated RBCs undergo agglutination. The occurrence of visible RBC agglutination serves as an indicator of a positive reaction, thereby confirming the presence of antibodies against *S. japonicum* in the test serum. IHAT has been utilized to assess the seroprevalence of *S. japonicum* across various definitive host species, including humans, cattle, and small ruminants such as goats and sheep (52–54). In a prior investigation, Yu et al. (52) compared the diagnostic performance of the Kato-Katz method, hatching test and IHAT for detecting human *S. japonicum* infection. Although IHAT demonstrated higher sensitivity relative to the Kato-Katz and hatching tests, its specificity was comparatively lower (51). Consequently, IHAT may not be appropriate as a standalone diagnostic tool for determining the prevalence of *S. japonicum* in clinical samples (52).

#### 3.3.2 Enzyme-linked immunosorbent assay (ELISA)

In recent years, enzyme-linked immunosorbent assay (ELISA) has been extensively applied for the detection of specific antibodies or antigens of specific pathogens, including *S. japonicum* (55). Liu et al. (56) identified two novel antigens, SjSAPLP4 and SjSAPLP5, which exhibited specific reactivity with serum samples obtained from diverse hosts infected with *S. japonicum*. Furthermore, ELISA assays developed utilizing these antigens demonstrated sensitivities and specificities of 98 and 100% for SjSAPLP4, and 96 and 100% for SjSAPLP5, respectively. These findings suggest that both SjSAPLP4 and SjSAPLP5 represent promising biomarkers for the development of ELISA-based diagnostic methods (56). Macalanda et al. (57) established an innovative ELISA approach targeting the cathepsin B of *S. japonicum*, which exhibited high sensitivity and specificity in identifying *S. japonicum* infection in both experimentally infected murine models and stool-confirmed human cases. Given its lack of cross-reactivity with serum samples positive for other parasitic infections, this ELISA technique appears to be a promising tool for the early diagnosis of *S. japonicum* infection (57).

In addition to the aforementioned candidates, Ren et al. (58) developed a double antibody sandwich ELISA utilizing an anti-Sj29 monoclonal antibody. The assay demonstrated detection sensitivities of 76.7% (23/30) in patients with acute *S. japonicum* infection, 54.5% (18/33) in those with chronic infection, and 50% (18/36) in individuals with advanced schistosomiasis (58).

#### 3.3.3 Others

In addition to ELISA and IHAT, various ELISA-based test strips have been developed for the detection of *S. japonicum*. Shen et al. (59) introduced an innovative fluorescence immunochromatographic assay strip designed to specifically identify antibodies against *S. japonicum*. Notably, this method exhibited a high concordance with ELISA results, as indicated by a Kappa value of 0.966. Furthermore, when compared to the Kato-Katz technique, the assay exhibited a sensitivity of 100% and a specificity of 96.34% (59).

Similarly, another research team developed a gold immunochromatographic assay strip incorporating the *S. japonicum* saposin protein, which demonstrated a statistically significant positive correlation with the corresponding ELISA assay based on the same protein (Pearson’s correlation coefficient, r = 0.7231) (60). Furthermore, this strip demonstrated stability after being stored at room temperature for 1 year, thereby confirming the reliability and consistency of the results (60). Additionally, the cost of the newly developed strip ranged from 1.5 to 4.0 USD per test, and demonstrated a sensitivity of 83.3% and a specificity of 100% in comparison with the Kato-Katz method (61).

## 4 The prevalence of *S. japonicum* among human and rodent populations in China

In recent years, Chinese government has carried out a series of effective measures aimed at eradicating schistosomiasis in definitive hosts. These interventions include replacing cattle with machinery, regulating specific areas for cattle and sheep breeding, and systematically removing animals that test positive for *S. japonicum*. Undoubtedly, these efforts have resulted in substantial advancements. For instance, the seroprevalence of *S. japonicum* infection in human populations in China declined from 34.8% in 1982 to 2.4% in 2020 (3). Moreover, the prevalence of this disease in definitive hosts such as cattle, sheep, dogs, pigs, and cats has been almost completely eliminated in China. However, this parasite remains widespread among human populations and wild rodents in certain regions of China. Therefore, this section primarily summarizes the prevalence of *S. japonicum* among humans and wild rodents in China.

### 4.1 Methods

#### 4.1.1 Literature research protocol

This review aims to provide a comprehensive overview of the epidemiological characteristics of *S. japonicum* infection among humans and wild rodents in China. The methodology was guided by the Preferred Reporting Items for Systematic Reviews and Meta-Analyses (PRISMA) framework (62). However, it is important to note that this review does not constitute a systematic review, but rather a narrative of available epidemiological data. Studies examining the prevalence of *S. japonicum* in humans or wild rodents in China, published from January 1, 2015 to April 1, 2026, were included. Relevant literatures were retrieved from six databases, including PubMed, Web of Science, Google Scholar, China National Knowledge Infrastructure, Wanfang, and Weipu.

The research methodology primarily involved the use of the following search terms: “*Schistosoma japonicum*” or “*S. japonicum*” or “Schistosomiasis” AND “prevalence” or “seroprevalence” or “epidemiological characteristics” or “epidemiology” AND “human” or “wild rodents” or “wild mice” AND “China” or “mainland China.” All retrieved articles were subsequently imported into EndNote software to facilitate reference management and the removal of duplicate literatures.

#### 4.1.2 Inclusion and exclusion criteria

Full-text articles were downloaded from these six databases for further analysis in this section, based on the following inclusion criteria: (1) the objective of the study was to evaluate the prevalence of *S. japonicum* infection in human populations or wild rodents in China. (2) the study reported both the total number of collected samples and the number of positive cases; (3) a clearly defined detection technique was provided; (4) the sample size exceeded 50 samples; (5) the geographic focus of the study was within regions of China; (6) the study employed a cross-sectional design; and (7) the study was performed between January 1, 2015 and April 1, 2026. Notably, this literature screening process was conducted independently by two authors (Fen Chen and Xi Hu) to ensure adherence to the eligibility criteria, and the final literature retrieved in this review was mediated by the third author (Lei Tan).

#### 4.1.3 Data integration and quantitative analysis

The inclusion of graphs and tables were included in this review aims to illustrate the transmission dynamics and prevalence rates of *S. japonicum* among humans and wild rodents in China. The aggregated prevalence of *S. japonicum* among different hosts was calculated utilizing the epidemiological calculator EpiTools software. Additionally, risk factors affecting the prevalence in human populations and wild rodents, encompassing regions, years, or species, were assessed through the application of the Chi-square test using SPSS version 24.0 (SPSS Inc., Chicago, IL, USA).

### 4.2 Literature search results

**Figure 2:**
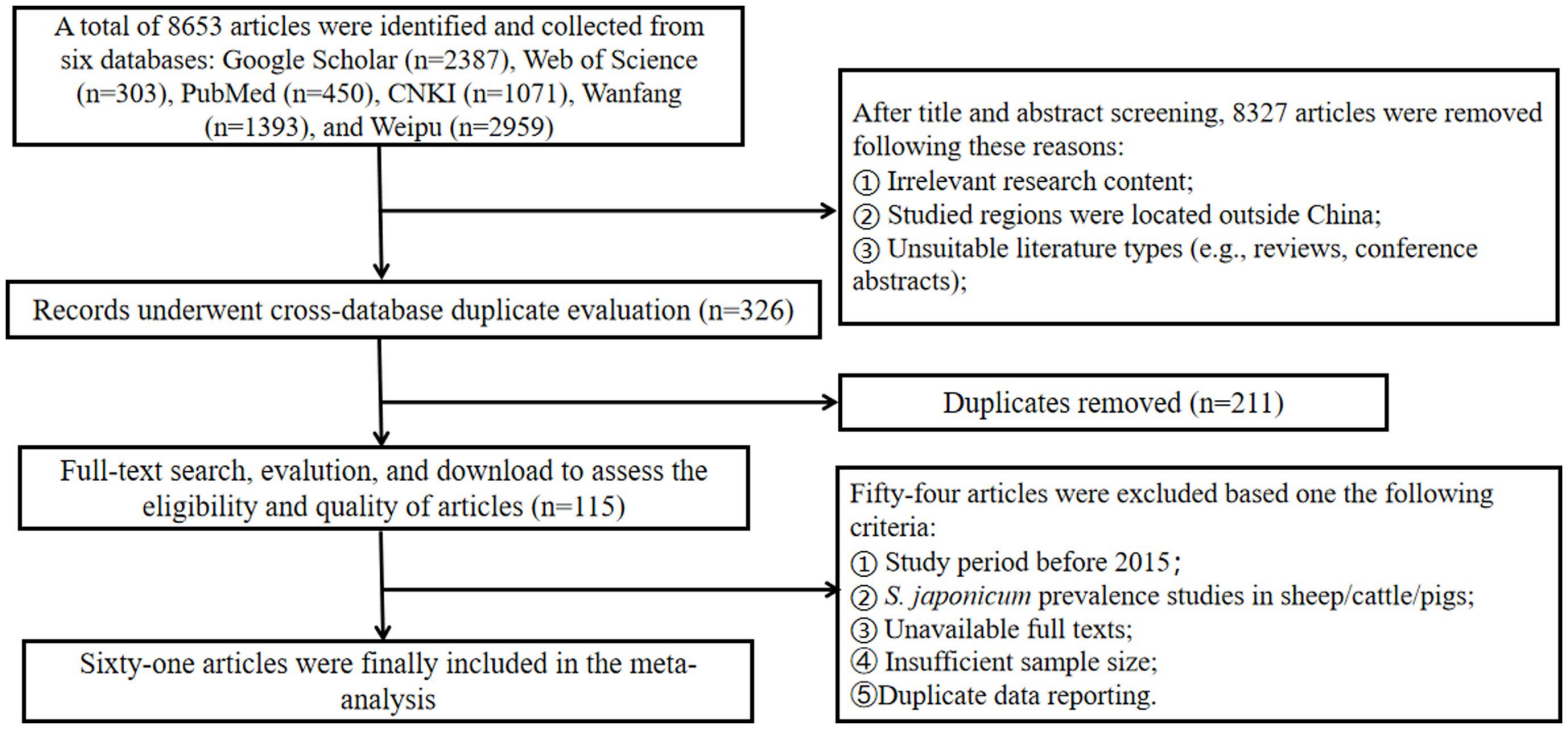
The flow chart of the selected research which examined the prevalence of *S. japonicum* across humans and wild rodents within China.

As illustrated in Figure 2, a total of 8,653 articles were retrieved from six Chinese and English databases, comprising 2,387 from Google Scholar, 303 from Web of Science, 450 from PubMed, 1,071 from CNKI, 1,393 from Wanfang, and 2,959 from Weipu databases. Following the removal of 211 duplicate records and an initial screening of 8,327 articles, 115 studies were selected for further evaluation. Ultimately, 61 articles satisfied all inclusion criteria and were included in this descriptive synthesis.

### 4.3 The aggregated prevalence of *S. japonicum* in humans in China

To present the most recent aggregated data regarding the prevalence of *S. japonicum* infection in humans within China, a total of 37 studies were included in this descriptive synthesis. The brief information of these articles was summarized in Supplementary Table 1 (63–99). Collectively, these studies examined a total of 46,910,186 human serum samples, among which 721,201 samples tested positive for specific antibodies against *S. japonicum*, yielding an average seroprevalence of 1.54% (95% CI: 1.53–1.54) based on aggregated data. Notably, numerous studies have examined the detection rate of *S. japonicum* eggs in fecal samples collected in China. However, the majority of these samples were obtained from individuals suspected of infection, or alternatively, no positive cases were detected among these randomly selected samples. Consequently, these studies have not undergone further detailed analysis.

**Figure 3:**
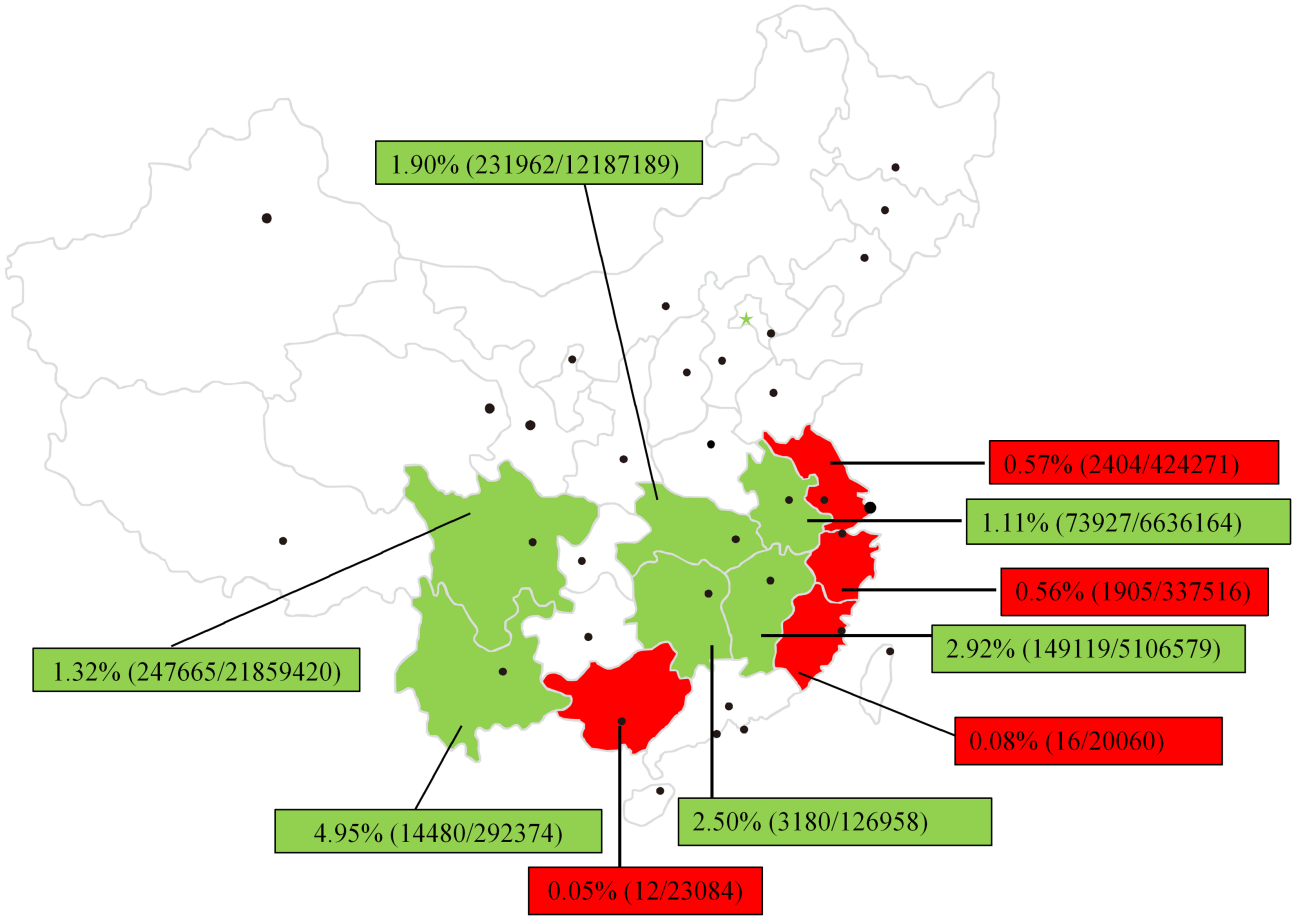
Summary of the seroprevalence of *S. japonicum* among human populations across different provinces in China from January 1, 2015 to April 1, 2026.

To further analyze potential risk factors influencing the seroprevalence of *S. japonicum* among human populations in China, investigations were mainly conducted across ten provinces and the findings were systematically summarized. As illustrated in Figure 3, the seroprevalence of *S. japonicum* infection within these provinces varied from 0.08% (16/20,060, 95% CI: 0.07–0.09) in Fujian Province to 4.95% (14480/292,374, 95% CI: 4.93–4.97) in Yunnan Province. Notably, four provinces (Fujian, Zhejiang, Guangxi, and Jiangsu Provinces) reported seroprevalence rates below 1.0%, whereas Jiangxi, Hunan, and Yunnan Provinces exhibited seroprevalence rates exceeding 2.0%. In fact, owing to the continuous prevention and control measures conducted in China, Fujian, Zhejiang, and Guangxi Provinces have been maintaining the elimination status of schistosomiasis for many years.

Regarding the seroprevalence of *S. japonicum* in China over different years (Table 2), the aggregated data showed that the seroprevalence decreased from 3.49% (73,982/2,133,190) in 2015 to 0.99% (18,852/1,906,879) in 2019, then increased to 2.20% (15,688/714,071) in 2020, followed by a gradual year-by-year decline thereafter. The average seroprevalence varies significantly between genders, with males exhibiting a higher prevalence of 2.19% (3,385/154,364) compared to females at 1.23% (1,445/117,358) (*p* < 0.01). Taking into account occupational variations, individuals involved in farming and fishing demonstrate elevated risks of infection by this parasite relative to other occupational groups, with mean seroprevalence of 1.97% (2,478/146,197) and 0.30% (349/115,003), respectively. Furthermore, a notable difference is observed between local and floating populations within the studied regions (*p* < 0.01), with average seroprevalence of 1.05% (4,805/458,094) and 0.67% (998/149,892), respectively.

**Table 2:** The aggregated seroprevalence of *S. japonicum* and its risk factors among Chinese human populations.

Factor	Category	No. of studies	No. of tested samples	No. of positive samples	Positive rate/% (95% CI)	*p*-value
Regions	Anhui	6	666164	73927	1.11 (1.08–1.14)	<0.01
	Guangxi	2	23084	12	0.05 (0.04–0.06)	-
	Hubei	7	12187189	231962	1.90 (1.89–1.97)	<0.01
	Jiangsu	9	424271	2404	0.57 (0.55–0.59)	0.8966
	Jiangxi	3	5106579	149119	2.92 (2.91–2.93)	<0.01
	Sichuan	5	21859420	247665	1.32 (1.318–1.323)	<0.01
	Yunnan	2	292374	14480	4.95 (4.93–4.97)	<0.01
	Zhejiang	2	337516	1905	0.56 (0.55–0.57)	Reference
	Hunan	3	126958	3180	2.50 (2.41–2.58)	<0.01
	Fujian	1	20060	16	0.08 (0.07–0.09)	-
Years	2015	17	2133190	73982	3.49 (3.47–3.51)	<0.01
	2016	23	2891859	70038	2.42 (2.40–2.44)	<0.01
	2017	23	2657754	62308	2.34 (2.32–2.36)	<0.01
	2018	24	2649429	51110	1.93 (1.91–1.95)	<0.01
	2019	23	1906879	18852	0.99 (0.98–1.00)	0.8341
	2020	17	714071	15688	2.20 (2.17–2.13)	<0.01
	2021	14	2330889	41673	1.79 (1.78–1.81)	<0.01
	2022	6	1252670	18729	1.50 (1.49–1.52)	<0.01
	2023	4	1205919	15748	1.31 (1.29–1.33)	<0.01
	2024	2	732045	6433	0.88 (0.86–0.90)	-
Population type	Local	11	458094	4805	1.05 (1.03–1.05)	<0.01
	Migrant	11	149892	998	0.67 (0.65–0.69)	
Gender	Man	4	154364	3385	2.19 (2.18–2.20)	<0.01
	Women	4	117358	1445	1.23 (1.21–1.25)	
Occupation	Farmers and fisherman	4	146197	2974	1.97 (1.89–2.04)	<0.01
	Others	4	115003	349	0.30 (0.27–0.33)	

### 4.4 The aggregated prevalence of *S. japonicum* in wild mice in China

Several species of wild mice have been identified across different regions of China. These rodent species serve as definitive hosts for *S. japonicum*, thereby contributing to the transmission and maintenance of this parasite among other carnivorous and omnivorous definitive hosts. In light of this, this section encompasses a total of 24 representative studies that examined the prevalence of *S. japonicum* infection among wild rodent populations across seven provinces and regions in China, the brief information of these articles was shown in Supplementary Table 2 (95, 100–122). As detailed in Table 3, this descriptive synthesis incorporates data from 14,381 wild rodents, among which 1,290 tested positive for *S. japonicum*, contributing to an aggregated prevalence of 8.97% (95% CI: 8.50–9.44). Moreover, the prevalence of this parasitic infection varied significantly across different provinces, ranging from a low of 0.03% (1/3514, 95% CI: 0.01–0.05) in Sichuan Province to a high of 27.54% (1,248/4,530, 95% CI: 26.24–28.84) in Anhui Province. In terms of the prevalence of *S. japonicum* among various rodent species in China, *Rattus norvegicus* demonstrated the highest infection rate at 37.44% (170/454, 95% CI: 33.00–41.89). Additionally, the mean infection rates of this parasite in *Rattus tanezumi*, *Apodemus agrarius*, *Niviventer confucianus*, *Mus musculus*, and other species were 17.25% (113/655, 95% CI: 14.36–20.14), 13.29% (82/617, 95% CI: 10.61–16.00), 13.56% (24/177, 95% CI: 8.51–18.60), 4.76% (1/21, 95% CI: 0.0–13.87), and 5.56% (2/36, 95% CI: 0.0–13.05), respectively.

**Table 3:** The aggregated prevalence of *S. japonicum* and its risk factors among wild rodents in China.

Factor	Category	No. of studies	No. of tested samples	No. of positive samples	Positive rate/% (95% CI)	OR (95% CI)	*P*-value
Regions	Anhui	10	4530	1248	27.54 (26.24–28.84)	82.8 (44.2–155.0)	< 0.01
	Hubei	3	3288	10	0.30 (0.11–0.48)	Reference	
	Hunan	1	76	7	9.21 (2.71–15.71)	33.3 (12.30–90.0)	< 0.01
	Jiangsu	5	458	14	3.06 (1.48–4.64)	10.3 (4.56–23.4)	< 0.01
	Jiangxi	1	172	5	2.91 (0.40–5.42)	6.72 (2.28–19.8)	< 0.01
	Sichuan	1	3514	1	0.03 (0.01–0.05)	–	–
	Yunnan	3	2343	5	0.21 (0.02–3.95)	1.07 (0.42–2.70)	0.893
Species	*Niviventer confucianus*	3	177	24	13.56 (8.51–18.60)	3.14 (0.4–24.5)	0.275
	*Mus musculus*	3	21	1	4.76 (0.0–13.87)	Reference	-
	*Apodemus agrarius*	5	617	82	13.29 (10.61–16.0)	3.07 (0.41–23.2)	0.278
	*Rattus norvegicus*	6	454	170	37.44 (33.0–41.89)	11.97 (1.59–90.2)	<0.05
	*Rattus tanezumi*	5	655	113	17.25 (14.36–20.14)	4.17 (0.55–31.4)	0.166
	Others	3	36	2	5.56 (0.0–13.05)	–	–

## 5 Perspective and further directions

The parasite *S. japonicum* has been endemic in China for over 1,000 years, leading to severe hepatic complications associated with portal hypertension (7). The average direct economic burden for patients in the advanced stages of schistosomiasis infection was estimated at 1,045 USD per year, while the average indirect economic burden was approximately 434 USD annually (123). Furthermore, infection with *S. japonicum* is closely linked to the development of several human malignancies, notably colorectal cancer and hepatocellular carcinoma (124, 125). In light of this, considerable efforts have been dedicated to the prevention and control of diseases caused by *S. japonicum* infection in China. Nevertheless, the disease continues to be endemic in specific regions of this country, posing a significant threat to the health of numerous human populations (126).

The prompt identification of infectious diseases is crucial for the timely implementation of preventive and control strategies, thereby mitigating adverse outcomes. Numerous diagnostic methodologies have been established for the detection of *S. japonicum* across diverse clinical contexts, which can be categorized into three primary groups: conventional morphological identification, serological methods, and molecular detection techniques. Although the three diagnostic categories have distinct technical features, their practical roles in surveillance and control programs differ considerably. Serological assays are most suitable for community-based screening and seroprevalence mapping due to their high throughput, simplicity, and rapid results. However, they cannot distinguish active from past infection because antibodies persist after parasite clearance, leading to overestimation of true prevalence, especially in low-endemic or elimination settings. For confirmatory diagnosis in seropositive individuals, traditional morphological methods (e.g., Kato-Katz, hatching test) offer high specificity but lack sensitivity for low-intensity infections, resulting in false negatives. Therefore, in low-prevalence settings, molecular techniques such as PCR or LAMP are increasingly recommended for confirmation due to their superior sensitivity and specificity. In conclusion, an integrated framework is proposed: serology for initial screening, molecular confirmation for seropositive individuals in low-transmission areas, and traditional microscopy for field surveillance in high-endemic regions.

It is noteworthy that the majority of studies have primarily utilized serological techniques to conduct preliminary assessments of the seroprevalence of *S. japonicum* infection. Subsequently, traditional morphological methods, including the Kato-Katz or hatching test, have been employed to confirm the presence of this parasite in individuals suspected of being infected. Notably, these traditional morphological techniques offer high specificity and are a good standard for confirmation. However, their low sensitivity, especially in low-intensity infections common in elimination settings, may lead to a high risk of false negatives. However, molecular detection techniques such as real-time PCR, RPA, LAMP, and RAA have not been extensively implemented for the diagnosis of *S. japonicum* infection in clinical practice. Several factors may underlie this phenomenon. First, the Kato-Katz technique and hatching test continue to serve as the primary diagnostic methods of schistosomiasis due to their relatively high specificity, operational practicality, and the absence of requirements for sophisticated equipment or complex interpretative criteria. Second, as revealed by a national survey examining the capacity building of schistosomiasis control institutes in China, it was observed that the majority of full-time schistosomiasis control personnel possess an associate or bachelor’s degree, these individuals may lack access to recently developed diagnostic techniques, including real-time PCR, RPA, and LAMP (126). Furthermore, over half of the surveyed laboratories did not possess the necessary experimental conditions to perform these advanced diagnostic procedures (127). Third, China conducts millions of tests annually to detect schistosomiasis infection (128). The adoption of molecular methods for monitoring the prevalence of this disease would entail higher costs. Furthermore, innovative detection methods, including RPA integrated with CRISPR-Cas12a technology and LAMP combined with the CRISPR-Cas12a system, demonstrate strong suitability for the site-specific identification of *S. japonicum* infections in diverse clinical settings. These techniques will have the potential for broader clinical application contingent upon a significant reduction in their associated costs. Similarly, though mNGS technology facilitates the unbiased identification of both known and novel pathogens, rendering its especially advantageous for the diagnosis of complex, polymicrobial, or uncommon infections. This approach is associated with several limitations, including substantial financial costs, intricate data analysis processes, and time-consuming, thus which is not suitable for investigating the prevalence of specific pathogens, including *S. japonicum*, in clinical settings.

Regarding the prevalence of *S. japonicum* infection within Chinese human populations, a descriptive synthesis of studies sourced from both English and Chinese databases demonstrated that this parasite is endemic in at least 10 provinces or regions across China. Aggregated data reveal an overall rate of 1.54% (721,201 positive cases out of 46,910,186 serum samples, 95% CI: 1.53–1.54). This result is consistent with a recent study (126), which reported an approximate seroprevalence rate of 1.80% based on 46,847,884 human serum samples collected from 12 provinces or regions in China between 2016 and 2023 (126). Moreover, the seroprevalence of *S. japonicum* exhibited considerable variability across the endemic regions, which may be attributed to multiple risk factors, including disparities in economic income, environmental toxicological factors, and climatic conditions among the provinces studied. According to the Gross Domestic Product ranking of China’s 31 provinces, Zhejiang, Jiangsu, Fujian, Hunan, and Hubei provinces are among the top 10. Higher income levels may enable greater investment in the prevention and control of schistosomiasis. Notably, the provinces of Hunan and Hubei are characterized by extensive lakes, marshlands, and coastal areas, which create favorable conditions for the transmission and persistence of schistosomiasis. Consequently, the prevalence of this disease in these regions is comparatively high.

The seroprevalence of *S. japonicum* within human populations was significantly affected by several risk factors, such as survey duration, population type, gender, and occupational status. Notably, the seroprevalence of *S. japonicum* declined from 2016 to 2019, then significantly rose in 2020, and then gradually decreased year by year. The marked increase in *S. japonicum* prevalence in 2020 may be attributed to the temporary reallocation or diversion of schistosomiasis control resources during the COVID-19 pandemic in China. With the effective prevention and control of COVID-19, the regular schistosomiasis eradication measures has been reinstated, resulting in a year-by-year decline in the disease’s prevalence.

Moreover, the local population exhibited a significantly greater seroprevalence relative to migrant individuals. Regarding the variation in seroprevalence across gender groups, the seroprevalence of *S. japonicum* among male individuals was approximately 1.8 times greater (95% CI: 1.69–1.91) than that observed in the female population. Moreover, individuals engaged in farming and fishing occupations exhibited a risk of *S. japonicum* infection that was more than fivefold higher (95% CI: 6.10–7.82) compared to those in other occupational categories. Several factors may contribute to these phenomenons: (1) Local inhabitants have historically lived in regions endemic for *S. japonicum*, resulting in increased and more frequent exposure to cercariae-contaminated water sources, such as rice paddies, rivers, and lakes, thereby elevating their risk of infection. In contrast, mobile populations typically reside in these areas for shorter durations and have comparatively limited contact with endemic water bodies, which corresponds to a reduced risk of infection. (2) Males are more frequently involved in outdoor and aquatic activities, including fieldwork, fishing, swimming, and irrigation, resulting in a substantially greater likelihood of skin exposure to contaminated water compared to females. Conversely, females tend to participate more in domestic tasks, indoor occupations, or activities situated at a greater distance from high-risk water sources, thereby experiencing reduced opportunities for exposure. (3) Farmers and fishermen work in environments that involve direct contact with infected water, such as paddy field cultivation, fishing, and salvaging aquatic plants. These activities expose them to water containing cercariae frequently and for long periods of time. In contrast, other professions (such as teachers, businessmen, artisans, etc.) have less exposure to infected water, and the risk of infection is significantly reduced.

In consideration of the characteristics of the aforementioned population groups, a series of comprehensive prevention and control strategies can be implemented. Priority should be given to targeted interventions for local residents with prolonged exposure to endemic areas, particularly males and individuals involved in agriculture, fishing, and animal husbandry. These interventions may include annual mass drug administration, such as oral praziquantel, administered prior to the transmission season. Additionally, the provision of protective equipment, such as waterproof boots and gloves, for farmers and fishermen is essential, alongside the promotion of health education aimed at minimizing contact with contaminated water sources.

From an environmental perspective, regular application of niclosamide to snail breeding sites, including rice paddies and irrigation ditches, should be conducted, coupled with modifications to irrigation systems to reduce suitable habitats for snail vectors. Furthermore, enhancing access to safe water supply infrastructure, such as the construction of wells and installation of tap water systems, can decrease residents’ dependence on natural water bodies. Establishing routine serological screening programs with prompt treatment for infected individuals is also critical to effectively disrupt the transmission cycle of *S. japonicum*.

**Figure 4:**
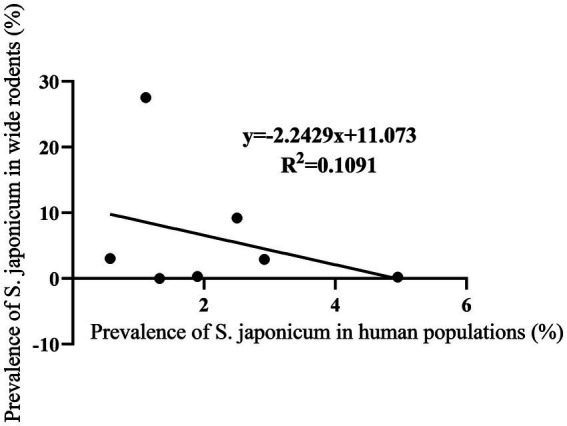
The concordance rate between the seroprevalence of *S. japonicum* in human populations and its prevalence in wild rodent populations was assessed across seven provinces in China.

Regarding the prevalence of *S. japonicum* infection among wild rodent populations in China, several key points have been emphasized: (1) Without considering the positive rates of serology and pathogenicity, the average infection rate in wild rodents is substantially higher than that observed in human populations. Firstly, human intervention strategies, such as pharmaceutical treatments and sanitation practices, have proven to be highly effective; however, these measures are not feasible for application to wild rodent populations, thereby leaving them entirely susceptible to infection; secondly, wild rodents serve as persistent reservoirs for the parasite. In contrast to humans and domesticated animals, they are not subject to control measures, thereby enabling them to perpetually contaminate the environment and sustain elevated levels of infection. (2) The prevalence of *S. japonicum* infection significantly ranged from various provinces. Among which Anhui and Hunan Provinces exhibited higher infection rates compared to other regions. Notably, these provinces contain various kinds of endemic zones that may contribute to the transmission of schistosomiasis in wild rodents, including lake and hill type areas. (3) In this review, a weak correlation was observed between the prevalence of *S. japonicum* in wild rodent populations and that in human populations, as evidenced by an R^2^ value of 0.1091 (Figure 4). From an epidemiological perspective, an R^2^ value of 0.1091 suggests that rodent prevalence accounts for only a minor proportion of the variability observed in human prevalence. This finding implies that alternative transmission routes, such as those involving cattle, environmental factors, or snails, are likely to have a more substantial influence. Nevertheless, this does not undermine the significance of wild rodents; rather, it underscores their function as independent maintenance hosts that necessitate direct monitoring, rather than being utilized as surrogate indicators of human risk.

This review provides a comprehensive summary of the current diagnostic methods employed for detecting *S. japonicum*, and the prevalence of this parasite in human and wild rodent populations in China. However, several limitations should be acknowledged. To prevent any potential misunderstanding, we explicitly state that the quantitative summaries provided in this document, including the overall seroprevalence estimates for humans and prevalence estimates for wild rodents, constitute descriptive summaries of the aggregated data. These findings are not the outcome of a formal meta-analytic approach, as no statistical pooling of effect sizes or application of meta-analytic models was conducted. First, significant heterogeneity was observed in the diagnostic approaches utilized across the studies. Certain investigations employed serological assays, which varied in terms of antigen selection and threshold values, whereas others relied on conventional morphological techniques such as the Kato-Katz method or hatching tests, or alternatively, molecular methodologies. Each diagnostic modality possesses unique sensitivity and specificity characteristics, and this methodological diversity poses challenges for the direct comparison of prevalence estimates between studies. Second, seroprevalence constitutes the principal outcome measure in the majority of the human studies reviewed, which possesses intrinsic limitations when employed as an indicator of active infection. Antibodies may remain detectable for extended periods, ranging from several months to years following parasite clearance, and serological assays are unable to differentiate between ongoing active infection and previous exposure. Consequently, seroprevalence estimates may overstate the actual prevalence of active infection, especially in settings characterized by low endemicity or those approaching elimination, where infection intensities tend to be minimal. Third, snails serve as the principal intermediate hosts for *S. japonicum*, playing a crucial role in the transmission of this parasite among both wild rodents as well as other definitive hosts (129). However, the prevalence of *S. japonicum* among snails is frequently overlooked. Fourth, wild rodents demonstrated a higher prevalence in China, data from certain endemic regions remain insufficient. Furthermore, the small sample sizes obtained from certain provinces, including Hunan and Jiangxi, may not provide an accurate representation of the actual prevalence within these regions. Likewise, the limited number of samples collected from specific wild rodent species may not reliably reflect the true prevalence in these species. Fifth, a variety of diagnostic approaches have been created to detect *S. japonicum,* while this review has not conducted meta-analysis to evaluate the diagnostic accuracy, specifically the combined sensitivity and specificity, of these technologies. Sixth, this review found that gender, population type, and occupation are significant risk factors affecting the prevalence of *S. japonicum* in human populations. However, owing to the lack of original data in the included studies, multiple linear analysis have not been performed to further assess these factors. In light of these gaps, future investigations should prioritize the improvement of snail surveillance through the application of molecular methodologies, broaden wild rodents sampling efforts in regions that are currently underrepresented, and undertake meta-analytical studies to assess the diagnostic performance of diverse technological approaches.

Overall, the observed human seroprevalence patterns, characterized by elevated risk among local residents, males, and individuals engaged in farming and fishing, underscore the influence of behavioral, occupational, and socio-environmental factors, with occupational exposure identified as the most significant predictor of infection. Despite prolonged implementation of control measures spanning multiple decades, challenges to the complete eradication of the disease persist. These include the underdetection of low-intensity infections attributable to limited application of molecular diagnostic techniques, the persistence of wild rodent reservoirs, and inadequate surveillance of snail intermediate hosts using advanced methodologies. These findings carry important implications for public health policy in China. Specifically, it is recommended that integrated diagnostic algorithms be adopted, employing serological assays for initial screening and molecular methods for confirmation in areas of low prevalence. Furthermore, the implementation of One Health approaches targeting humans, rodent reservoirs, and snail vectors is essential. Control programs should be designed with built-in resilience to maintain effectiveness during public health emergencies. Emphasis should be placed on high-risk groups, notably local male farmers and fishermen, by implementing interventions tailored to their specific occupations. Additionally, strengthening laboratory infrastructure at the county level and delivering extensive training in molecular diagnostic techniques are essential for progressing toward elimination objectives.

## 6 Conclusion

In summary, *S. japonicum* continues to be endemic in certain regions of China, with wild rodents playing a key role as reservoir hosts. Recent diagnostic improvements, especially molecular tools based on CRISPR/Cas technology, provide greater sensitivity and are suitable for field use, although traditional techniques like Kato-Katz are still commonly used. Epidemiological studies show a human seroprevalence of 1.54%, while infection rates in wild rodents are much higher at 8.97%, highlighting the importance of including wildlife in monitoring efforts. To meet the national goal of eliminating the disease by 2030, it is essential to combine sensitive diagnostic methods with thorough management of reservoir hosts.

## Supplementary material

Supplementary Table 1 Summary of the studies that investigated the seroprevalence of *S. japonicum* among human populations in China from January 1, 2015 to April 1, 2026.

Supplementary Table 2 Summary of 24 studies investigating the prevalence of *S. japonicum* infection among wild rodents in China.
