# Supplementary material for: A mini review on advances in diagnostic techniques for Schistosoma japonicum detection and its epidemiological features among humans and wild rodents in China
Source: Front Vet Sci. 2026 May 28;13:1857648. doi: 10.3389/fvets.2026.1857648 (PMC13253484; doi:10.3389/fvets.2026.1857648)
Supplement: Supplementary Table 1 — Summary of the studies that investigated the seroprevalence of S. japonicum among human populations in China from January 1, 2015 to April 1, 2026. [file Table_1.docx]

| **No** | **Period** | **Investigated Province** | **No. of tested samples** | **No. of positive samples** | **Positive rate/%** | **Reference** |
| --- | --- | --- | --- | --- | --- | --- |
| 1 | 2001-2021 | Sichuan | 5641 | 17 | 0.3 | [63] |
| 2 | 2001-2024 | Zhejiang | 103101 | 717 | 0.17 | [64] |
| 3 | 2002-2021 | Jiangxi | 451238 | 15837 | 3.51 | [65] |
| 4 | 2004-2021 | Yunnan | 280454 | 13841 | 4.94 | [66] |
| 5 | 2011-2019 | Jiangsu | 34822 | 193 | 0.55 | [67] |
| 6 | 2004~2021 | Jiangsu | 26326 | 6 | 0 | [68] |
| 7 | 2004~2022 | Hubei | 536466 | 6530 | 1.22 | [69] |
| 8 | 2004~2023 | Zhejiang | 234415 | 1188 | 0.51 | [70] |
| 9 | 2012~2021 | Sichuan | 5917 | 163 | 2.75 | [71] |
| 10 | 2012~2022 | Sichuan | 354746 | 2567 | 0.72 | [72] |
| 11 | 2013~2022 | Hubei | 740483 | 11072 | 1.5 | [73] |
| 12 | 2015~2019 | Anhui | 5571 | 284 | 5.1 | [74] |
| 13 | 2015~2020 | Hubei | 54458 | 468 | 0.86 | [75] |
| 14 | 2015~2024 | Hubei | 10744800 | 213400 | 1.99 | [76] |
| 15 | 2015~2023 | Sichuan | 20545155 | 232157 | 1.13 | [77] |
| 16 | 2016~2020 | Anhui | 6362300 | 69900 | 1.1 | [78] |
| 17 | 2016~2020 | Jiangsu | 114890 | 1033 | 0.9 | [79] |
| 18 | 2016~2020 | Jiangsu | 19910 | 286 | 1.44 | [80] |
| 19 | 2016~2020 | Jiangsu | 122376 | 307 | 0.25 | [81] |
| 20 | 2020~2024 | Anhui | 119455 | 1012 | 0.84 | [82] |
| 21 | 2016~2023 | Jiangxi | 4570976 | 129910 | 2.84 | [83] |
| 22 | 2016~2020 | Jiangsu | 90335 | 361 | 0.4 | [84] |
| 23 | 2020~2024 | Guangxi | 20473 | 1 | 0 | [85] |
| 24 | 2020~2024 | Hubei | 74009 | 222 | 0.3 | [86] |
| 25 | 2020 | Hubei | 20520 | 184 | 0.9 | [87] |
| 26 | 2021~2022 | Jiangsu | 6196 | 13 | 0.21 | [88] |
| 27 | 2021 | Sichuan | 947961 | 12761 | 1.35 | [89] |
| 28 | 2023-2024 | Yunnan | 11920 | 639 | 5.36 | [90] |
| 29 | 2023 | Jiangsu | 7273 | 158 | 2.17 | [91] |
| 30 | 2020 | Anhui | 20130 | 298 | 1.48 | [92] |
| 31 | 2015~2019 | Anhui | 92466 | 1821 | 1.97 | [93] |
| 32 | 2015~2021 | Anhui | 36242 | 612 | 1.79 | [94] |
| 33 | 2018~2020 | Jiangsu | 2143 | 47 | 2.19 | [95] |
| 34 | 2015~2019 | Hunan | 85529 | 2198 | 2.57 | [96] |
| 35 | 2016~2020 | Hunan | 41296 | 904 | 2.19 | [97] |
| 36 | NA | Hunan | 133 | 78 | 58.65 | [98] |
| 37 | 2015~2019 | Fujian | 20060 | 16 | 0.8 | [99] |
