# Supplementary material for: A mini review on advances in diagnostic techniques for Schistosoma japonicum detection and its epidemiological features among humans and wild rodents in China
Source: Front Vet Sci. 2026 May 28;13:1857648. doi: 10.3389/fvets.2026.1857648 (PMC13253484; doi:10.3389/fvets.2026.1857648)
Supplement: Supplementary Table 2 — Summary of 24 studies investigating the prevalence of S. japonicum infection among wild rodents in China. [file Table_2.docx]

| **No** | **Period** | **Investigated Province** | **No. of tested samples** | **No. of positive samples** | **Positive rate/%** | **Reference** |
| --- | --- | --- | --- | --- | --- | --- |
| 1 | 2018 | Anhui | 376 | 91 | 24.2 | [100] |
| 2 | 2020-2022 | Anhui | 922 | 120 | 13.02 | [101] |
| 3 | 2016~2020 | Jiangsu | 215 | 14 | 6.51 | [102] |
| 4 | 2022~2023 | Yunnan | 1006 | 5 | 0.5 | [103] |
| 5 | 2024~2025 | Yunnan | 891 | 0 | 0 | [104] |
| 6 | 2021 | Anhui | 115 | 54 | 46.96 | [105] |
| 7 | 2017 | Hunan | 76 | 7 | 9.21 | [106] |
| 8 | 2018-2020 | Anhui | 333 | 89 | 26.73 | [107] |
| 9 | 2018-2019 | Anhui | 199 | 45 | 22.61 | [108] |
| 10 | 2021 | Anhui and Jiangxi | 237 | 121 | 50.5 | [109] |
| 11 | 2017-2019 | Jiangsu | 132 | 0 | 0 | [110] |
| 12 | 2021-2022 | Anhui | 326 | 36 | 11.04 | [111] |
| 13 | 2018 | Yunnan | 446 | 0 | 0 | [112] |
| 14 | 2018 | Jiangxi | 172 | 5 | 2.91 | [113] |
| 15 | 2010-2018 | Sichuan | 3514 | 1 | 0 | [114] |
| 16 | 2019-2023 | Hubei | 361 | 0 | 0 | [115] |
| 17 | 2015-2018 | Hubei | 2185 | 10 | 0.46 | [116] |
| 18 | 2022 | Anhui | 1703 | 678 | 39.81 | [117] |
| 19 | 2016-2018 | Anhui | 104 | 0 | 0 | [118] |
| 20 | 2016-2020 | Jiangsu | 215 | 14 | 6.51 | [119] |
| 21 | 2020 | Jiangsu | 8 | 0 | 0 | [120] |
| 22 | 2017-2019 | Hubei | 742 | 0 | 0 | [121] |
| 23 | 2021-2023 | Jiangsu | 26 | 0 | 0 | [122] |
| 24 | 2018-2020 | Jiangsu | 77 | 0 | 0 | [123] |
